# Investigation of Flavor-Forming Starter *Lactococcus lactis* subsp. *lactis* LDTM6802 and *Lactococcus lactis* subsp. *cremoris* LDTM6803 in Miniature Gouda-Type Cheeses

**DOI:** 10.4014/jmb.2004.04004

**Published:** 2020-06-05

**Authors:** Hye Won Lee, In Seon Kim, Bum Ju Kil, Eunsol Seo, Hyunjoon Park, Jun-Sang Ham, Yun-Jaie Choi, Chul Sung Huh

**Affiliations:** 1 Graduate School of International Agricultural Technology, Seoul National University, Pyeongchang 25354, Republic of Korea; 2 Department of Agricultural Biotechnology, Seoul National University, Seoul 08826, Republic of Korea; 3 Institute of Green-Bio Science and Technology, Seoul National University, Pyeongchang 25354, Republic of Korea; 4 WCU Biomodulation Major and Center for Food and Bioconvergence, Seoul National University, Seoul 08826, Republic of Korea; 5 Research Institute of Agriculture and Life Science, Seoul National University, Seoul 08826, Republic of Korea; 6 National Institute of Animal Science, Rural Development Administration, Wanju 55365, Republic of Korea

**Keywords:** Gouda cheese, Lactococcus, starter culture, cheese flavor

## Abstract

Lactic acid bacteria (LAB) play an important role in dairy fermentations, notably as cheese starter cultures. During the cheese production and ripening period, various enzymes from milk, rennet, starter cultures, and non-starter LABs are involved in flavor formation pathways, including glycolysis, proteolysis, and lipolysis. Among these three pathways, starter LABs are particularly related to amino acid degradation, presumably as the origins of major flavor compounds. Therefore, we used several enzymes as major criteria for the selection of starter bacteria with flavor-forming ability. *Lactococcus lactis* subsp. *lactis* LDTM6802 and *Lactococcus lactis* subsp. *cremoris* LDTM6803, isolated from Korean raw milk and cucumber kimchi, were confirmed by using multiplex PCR and characterized as starter bacteria. The combinations of starter bacteria were validated in a miniature Gouda-type cheese model. The flavor compounds of the tested miniature cheeses were analyzed and profiled by using an electronic nose. Compared to commercial industrial cheese starters, selected starter bacteria showed lower pH, and more variety in their flavor profile. These results demonstrated that LDTM6802 and LDTM6803 as starter bacteria have potent starter properties with a characteristic flavor-forming ability in cheese.

## Introduction

Milk and dairy products, including cheeses, are important components of the human diet and play an essential role in meeting nutritional requirements [1]. Gouda cheese is a washed-curd Dutch cheese that is traditionally produced from bovine milk [2-4]. It is soaked in a brine solution and ripened for 1 to 20 months, which improves the taste [5]. The Code of Federal Regulations of the United States specifies a maximum moisture content of 45% by weight and a minimum 46% fat content on a dry weight basis for Gouda cheeses.

Starter cultures used in the production of cheeses are typically composed of mesophilic lactic acid bacteria (LAB) having acidification activity, proteolytic activity, and flavor production. [6, 7]. In particular, the Gouda- type cheeses are primarily made from *Lactococcus lactis* subsp. *lactis* and *L. lactis* subsp. *cremoris,* which are principal acid producers [8]. In addition, *L. lactis* subsp. *lactis* biovar *diacetylactis* and/or *Leuconostoc* spp. are secondary microflora that ferment citrate with the production of CO2, diacetyl, and acetate [9].

As the cheese market has grown, consumers and producers have focused on new types of cheese with unique flavor characteristics [10]. Cheese flavor is a result of complex processes originating from a combination of microbiological, biochemical, and technological aspects. As cheese ripens, the starter culture produces the flavor compounds through carbohydrates fermentation, casein proteolysis, milk fat lipolysis, and non-enzymatic reactions [11-13]. Lactose is converted to lactic acid and acetic acid, which decrease the pH during cheese ripening. The production of these acids contributed to the fresh acidic flavor of the cheese. Moreover, lactic acid can be further metabolized into various flavor compounds, including ethyl esters. The citrate can be converted to acetic acid, diacetyl, acetoin, and butane-2,3-dione [14]. In addition, *Lactococcus* species produce amino acids from central metabolism and have a well-characterized cell envelope-associated protease that hydrolyzes casein to amino acids [15]. Intracellularly, most amino acids can be converted to α-keto acids by aminotransferases. The central intermediate α-keto acids can be converted to aldehydes, hydroxyl acids, and CoA-esters by decarboxylase and dehydrogenase [16]. Methyl ketones, secondary alcohols, esters, and lactones can be produced from lipolysis of milk fat [17].

New starter strains of LAB isolated from different milk environments and fermented foods may show good potential for developing improved cheese flavor [18, 19]. This study focuses on the flavor-forming ability of lactococci strains originated from Gangwon Province in Korea for production of the Gouda-type cheese. To validate the potential of the selected strains as Gouda cheese starters, we confirmed the enzyme distributions related to the flavor compounds by specific polymerase chain reaction (PCR) and analyzed the aroma profiles of miniature cheese models by electronic nose.

## Materials and Methods

### Bacterial Strains and Culture Conditions

*Lactococcus lactis* subsp. *lactis* LDTM6802 (KCTC18611P) and *Lactococcus lactis* subsp. *cremoris* LDTM6803 (KCTC18612P) were isolated from cucumber kimchi and raw milk products from Gangwon Province, South Korea. Collected kimchi samples were serially diluted 10-fold using 0.85% NaCl solution (Sigma-Aldrich, USA) and homogenized. Serial dilutions were plated on M17 (Difco, USA) supplemented with 0.5% lactose (GM17) (Difco) agar for lactococci isolation. Inoculated plates were incubated at 30°C under aerobic conditions for 48 h. The strains were deposited in the Korean Collection for Type Cultures (KCTC). We used the commercial starter culture CHN-11 (Chr. Hansen, Denmark), which contains *Lactococcus lactis* subsp. *lactis*, *Lactococcus lactis* subsp. *cremoris*, *Lactococcus lactis* subsp. *lactis* biovar *diacetylactis*, and *Leuconostoc* subsp. *mesenteroides*. We isolated *Lactococcus lactis* subsp. *cremoris* (CHN-11-3), *Lactococcus lactis* subsp. *lactis* biovar *diacetylactis* (CHN- 11-1), and *Leuconostoc* subsp. *mesenteroides* (CHN-11-48) from CHN-11 starter, as confirmed by 16S rRNA gene sequence analysis. *Lactococcus lactis* subsp. *lactis* IL1403 (KCTC3115) was used as a type strain. These strains were cultured in M17 (Difco) supplemented with 0.5% lactose (GM17) at 30°C under aerobic conditions and stored in 20% glycerol (Sigma-Aldrich) at -80°C.

### Acidification and Coagulant Activity

Each strain was inoculated at 1% (v/v) in sterilized 10% skim milk (Oxoid, UK) under aerobic conditions at 30°C for 48 h. The acidification activity was measured by using the Orion Star A211 pH-Meter (Thermo Scientific, USA) after 6 and 48 h incubation. The coagulant activity was evaluated by the appearance of a coagulum of 10% skimmed milk, after manually tilting the tube [20].

### Multiplex PCR

The multiplex PCR assay was used to detect the genes for enzymes involved in flavor-forming pathways. The primers were designed manually by using the Primer-BLAST tool of National Center for Biotechnology Information (NCBI, USA) and were commercially obtained (Bioneer, Korea). Amplification was performed on a PCR system in a 20 μl reaction mixture consisting of i-Taq 2X PCR Master Mix Solution (iNtRON, Korea) with one colony of each streaking plate. The following PCR program was used for 30 cycles: 30 sec at 95°C, 20 sec at 41- 43°C, and 2.2 min at 72°C. The amplified products of each group were run on 1.5% agarose in 1× TAE buffer at 70 v for 40 min.

### Glutamate Dehydrogenase (GDH) Assay

Each strain was inoculated at 1% (v/v) in 10 ml of media and incubated at 30°C under aerobic conditions for 18 h. The cells were harvested by centrifugation, washed twice with phosphate-buffered saline (Mediatech Inc., USA), and added to microtubes containing 2.0 g of Zirconia/silica beads (0.1 mm dia., BioSpec Products, USA). Each of the cell-free extracts (CFEs) was obtained in the following manner: two cycles of 30-sec disruption at the highest speed of the disruptor (Mini-Beadbeater-16, BioSpec Products) and 1 min of cooling on ice. The CFEs were stored at -20°C until further use [21].

The GDH activities of the CFEs were analyzed by using a commercial colorimetric glutamic acid assay (R- Biopharma, Germany). The reaction mixture contained 80 μl of distilled water, 80 μl of potassium phosphate/ triethanolamine buffer (pH 8.6), 40 μl of 100 mM of L-glutamic acid (Sigma-Aldrich), 40 μl of iodonitrotetrazolium, and 40 μl of NAD•diaphorase (600 μl in total). After the addition of 60 μl of CFE to the reaction mixture, 200-μl aliquots of the mixture were immediately distributed to each of 3 wells and incubated for 3 h at 30°C and the absorbance was measured at 492 nm [8].

### Growth and Autolysis Abilities

Growth curves were obtained for selected strains cultivated at 30°C with 1% (v/v) inoculation in GM17. The bacterial concentrations were determined by measuring the absorbance at 600 nm by using a spectrophotometer. In addition, the selected strain was incubated at 30°C with 1% (v/v) inoculation in GM17 and the pH change over time was measured by pH meter. API CHL 50 and API ZYM (BioMérieux, France) were used for carbohydrate fermentation and enzymatic profiling, respectively. The API tests were performed according to the manufacturer’s instructions.

The rate of cell autolysis was measured according to the method of El Soda *et al*. [22]. Briefly, the different cell cultures were harvested by centrifugation (8,000 g, 15 min, 4°C) and washed twice with 0.85% NaCl. The obtained pellet was then resuspended in 0.01 M phosphate buffer (pH 5.5) containing 0.5 M NaCl. The cell suspension was initially adjusted at an optical density of 0.9 to 1.0 at 600 nm by using spectrophotometer.

### Manufacture of Miniature Cheese Models

The miniature cheese models were manufactured according to Hynes *et al*. [23]. Three miniature cheeses, two control groups and one test group were produced by different starter cultures. The starter strains were inoculated into sterile 10% skim milk. After 18 h of incubation at 30°C, each culture was mixed as control 1 (CHN-11-3, IL1403), control 2 (CHN-11-3, IL1403, CHN-11-1, and CHN-11-48), and test (LDTM6802 and LDTM6803) groups. Liquid rennet extract of bovine origin (STD PLUS 290, Chr. Hansen) was used. The cheeses were produced in bottles with 400 ml of milk (30°C), inoculated starter (2%, v/v), and 80 μl of rennet. The bottles were immediately covered, manually inverted three times, and finally incubated in a water bath at 30°C. After coagulation, the bottles were maintained in a water bath. The coagulum was cut with sterile disposable steel tools. The mixture of whey-curd particles was agitated for 20 min in a mechanical stirrer by inversion of the bottles. Approximately 40% (160 ml) of the whey was discarded and replaced by an equal volume of sterile water at 30°C. Curd washing was completed by stirring for 10 min as described above.

The bottles were centrifuged at 320 g for 10 min at room temperature to remove most of the aqueous phase. Subsequently, the curd was transferred to sterile cylindrical 150 ml recipients, and re-centrifuged at 1,400 g for 1 h. The whey was then discarded and the curd was inverted in the same container. A final centrifugation was performed at 1,400 g for 30 min. The miniature cheeses were stored in sterile containers and salted by pouring 32 ml of sterile saturated brine (330 g/L of NaCl, pH adjusted to 5.4) at 10°C into the same containers. After 5 min, the brine was removed, and the miniature cheeses were ripened at 10°C for 28 days [24].

### Evaluation of Flavor Profile by Electronic Nose

The Heracles II Analyzer/GC E-Nose (Alpha M.O.S, France), consisting of a dual-column flash gas- chromatograph coupled with a Combi PAL Auto-Sampler System (CTC Analytics AG, Switzerland), was used. The analysis was performed according to the manufacturer’s instructions (Alpha M.O.S). Gas samples from the headspace after pre-incubation for 20 min at 70°C were collected from each 10 ml vial containing 1 g of cheese and delivered by the auto-sampler to an inlet injector in which the compounds were flash evaporated. After passing through a pre-concentration TENAX adsorbent trap, the volatiles carried by the hydrogen are equally sent to two capillary chromatographic columns. The two columns have different polarities (one column was apolar, GC1#: DB-5, and the other column had medium polarity, GC#2: DB-WAX), and various volatile compounds were simultaneously separated on both of these columns and detected by flame ionization detectors (FID) working in parallel. The following analytical conditions for automatic gas injection with a 2.5-ml HS syringe from the headspace were used: quantity of sample: 1 g in a 10 ml vial, sample incubation 20 min at 70°C (agitation speed 500 rpm, agitation on 5 sec, agitation off 2 sec, flushing time 90 sec), syringe temperature 80°C, fill speed 500 μl /sec, injection volume 5,000 μl, injection speed 125 μl/s, injection temperature 200°C, injection pressure 10 kPa, injection time 45 sec, trap temperature 50°C, trap pre-heating temperature 35°C, column temperature program 50°C (initial, hold time 2 sec) up to 250°C (21 sec) with an increment of 1°C/sec to 80°C and an increment of 3°C/sec to 250°C, with a detector temperature of 260°C. The two chromatograms were analyzed by Ar°ChemBase specialized software (Alpha M.O.S) for sensory descriptions.

### Statistical Analysis

All data were statistically analyzed using Graph-Pad Prism software version 5.01 (Graph-Pad Software, USA). The statistical significance of the differences was determined by one-way analysis of variance (ANOVA) followed by Tukey’s post hoc test. Differences were considered significant at *p* < 0.05.

## Results

### Analysis of Lactic Acid Bacteria for Cheese Starter

The main role of starter bacteria in cheese manufacturing is to provide coagulant activity and rapid acidification. CHN-11-1, CHN-11-3, LDTM6802, and LDTM6803 strains had positive coagulant activity at 24 and 48 h (Table 1). According to the acid production monitoring after 6 and 48 h, the Chr. Hansen starter strains and control strains showed a pH value from 6.05 to 6.15 at 6 h and from 4.39 to 5.72 at 48 h. That means the CHN- 11-1 and CHN-11-3 strains played roles as a starter culture for the acidification of milk. The LDTM6802 and LDTM6803 strains showed higher acid production than these strains at 6 h (Table 1).

**Fig. 1:**
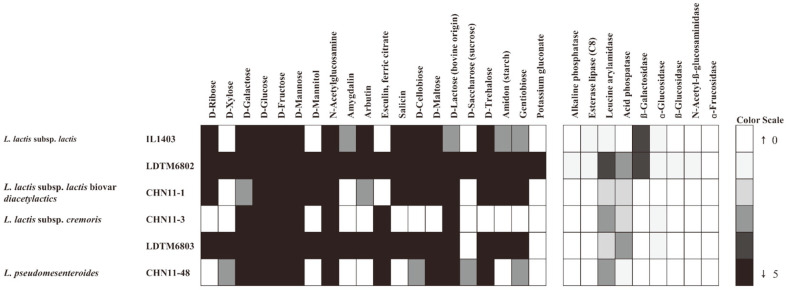
The carbohydrate fermentation and enzymatic patterns of selected *L. lactis* subsp. *lactis* strains and *L. lactis* subsp. *cremoris* strains were assessed compared to control strains by API CHL 50 and API ZYM kits. In the API CHL 50, black and white represent positive and negative activity, respectively, and gray means weak activity. The API ZYM are shown in a black-white color scale (0 to 5) according to the manufacturer’s instructions.

To investigate the flavor-forming ability of lactic acid bacteria by carbohydrate, protein, and fat fermentation, LDTM6802 and LDTM6803 were analyzed by multiplex PCR and GDH activity (Table 1). The CHN-11-1 and IL1403 strains encoded all target genes except *citP*, whereas CHN-11-3 encoded only *bcaT,* and CHN-11-48 encoded none of the target genes. The LDTM6802 encoded *adhE, bcaT, araT, estA*, and *adh* genes, and LDTM6803 encoded *bcaT* and *kdcA* genes (Table 1). The level of NAD-dependent GDH was also analyzed (Table 1). The LDTM6802 had a significant level of GDH activity compared to that of IL1403, CHN-11-1, and CHN-11-3. In addition, the GDH activity of LDTM6803 showed higher activity than that of IL1403, CHN-11-1, and CHN-11-3. Furthermore, to confirm the production ability of the flavor compounds, the carbohydrate fermentation and enzymatic patterns were assayed with the Chr. Hansen starter and selected strains (Fig. 1). The LDTM6802 and LDTM6803 strains had the strongest carbohydrate fermentation activity, and the LDTM6802 showed a variety of enzymatic patterns compared to those of IL1403, CHN-11-1, CHN-11-3, and CHN-11-48. Overall, the selected strains showed similar genotypic characteristics and better phenotypic characteristics than the Chr. Hansen starters.

### Growth and Fermentation Properties

**Fig. 2:**
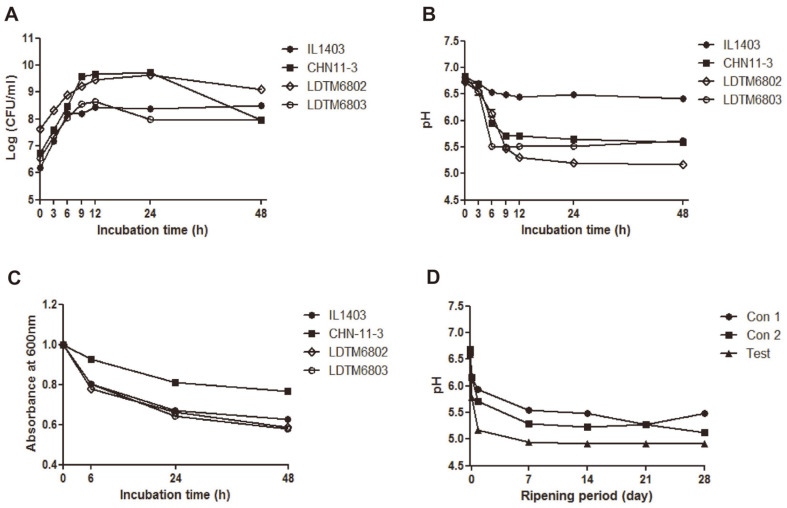
Growth and fermentation properties of the tested strains and pH change of miniature cheese products. (**A**) bacterial growth curves. (**B**) pH change of the strains. (**C**) autolysis activity. (**D**) pH change of the miniature cheese products during ripening. Data are presented as means ± SEMs.

Autolysis can accelerate the production of metabolites by proteolysis and lipolysis through an early release of intracellular enzymes during cheese ripening. To confirm the growth pattern and enzyme release ability, growth, pH curves and autolysis were analyzed in this study. All strains reached their maximum growth within 24 h and *L. lactis* subsp. *lactis* strains were relatively stable up to 48 h compared to *L. lactis* subsp. *cremoris* strains (Fig. 2A). All strains except IL1403 showed a sharp decrease in pH during 12 h incubation, while LDTM 6802 had the lowest pH (Fig. 2B). In addition, LDTM6802 and LDTM6803 strains had high autolysis activity compared to that of control IL1403 and CHN-11-3 (Fig. 2C). The pH of the control and test miniature cheeses dropped dramatically at 1 week of ripening, and after that the control cheeses decreased above pH 5, while test cheese dropped to below pH 5. In addition, the test cheese had a stable pH from the beginning of the ripening period (Fig. 2D).

### Flavor Compound Analysis of Miniature Gouda-Type Cheese

**Fig. 3:**
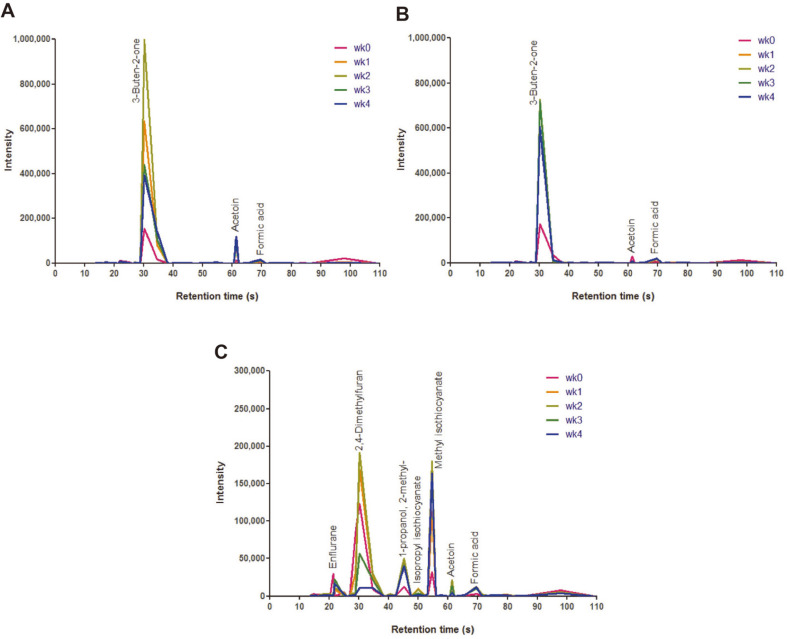
Measurement of representative flavor compounds of 10°C ripened miniature cheese products. (**A**) control 1. (**B**) control 2. (**C**) test cheese.

To evaluate the flavor of miniature Gouda-type cheese by the selected strains, control and test miniature cheeses were manufactured. All miniature cheeses were ripened at 10°C for 4 weeks and analyzed using an electronic nose. The test miniature cheeses were found to have more varied flavor profiles than the control miniature cheeses. After 4 weeks, the ripened cheeses of the control and test groups at 10°C were detected with some flavor compounds, such as acetoin, butane-2,3-dione, ethanol, formic acid, 1,3-dimethylcyclohexane, and 3-buten-2- one (Table 2). Among these compounds, the main peak of 3-buten-2-one, a small peak of acetoin, and formic acid were observed in the control group for 4 weeks. (Figs. 3A and 3B). The intensity of the compound peaks varied with the length of the ripening period. In control 1, 3-buten-2-one was the highest at 2 weeks of ripening and then decreased. Also, 3-buten-2-one in control 2 was highest at 3 weeks and then decreased. On the other hand, the ripened cheese of the test group showed additional peaks of enflurane, isopropyl isothiocyanate, methylbutanone, methyl isothiocyanate, 2-methyl-1-propanol, and 2,4-dimethylfuran, but did not show 3-buten-2-one. At 2 weeks of ripening, 2,4-dimethylfuran increased and decreased, and at 4 weeks, methyl isothiocyanate increased (Fig. 3C). Therefore, *L. lactic* subsp. *lactis* LDTM6802 and *L. lactis* subsp. *cremoris* LDTM6803 produced more flavor compounds compared to control cheese starter strains on the last ripening day.

The results demonstrated that the cheeses have buttery, sweet, savory, alcoholic, and perfumery flavors, with flavor compounds such as acetoin, butane-2,3-dione, 3-buten-2-one, formic acid, ethanol, and 1,3-dimethylcyclohexane (Table 2). In addition, the cheeses produced with LDTM6802 and LDTM6803 have floral, vegetable, bitter, camphor, and almond flavors with flavor compounds such as 3-formythiophene, 2-methyl-1- propanol, methylbutanone, and furfural.

## Discussion

Cheese and cheese starter culture-related studies are constantly being conducted worldwide [25-28]. One of the most important cheese starter abilities is curd formation in milk by acid production [18, 29]. Rapid growth, acidification ability, autolysis ability, carbohydrate fermentation pattern, and enzymatic profile are also selection criteria [7]. By using multiplex PCR assay, the *adhE, bcaT, araT, estA, adh, citP*, and *kdcA* genes were detected in the selected strains. LDTM 6802 had these genes, but did not have *citP*. The *citP* gene was not detected in the selected bacteria, but the *kdcA* gene was found in LDTM6803. The absence of the *citP* gene in isolates was consistent with the findings of previous studies [9, 30]. According to Liu *et al*. [31], the *kdcA* enzyme activity was only found in non-dairy lactococcal strains. In addition, the GDH activity, carbohydrate fermentative pattern, pH stability, and enzymatic profiling results showed selected probiotic strains can utilize more than the control strain.

Cheese flavors are produced through a complex process. According to the Component Balance Theory of Cheese Flavor, a cheese flavor result is an accurate balance and concentration of a wide variety of volatile flavor compounds [11]. The flavor of cheese varies depending on the cheese type, and even the same type of cheese varies in flavor depending on the starter bacteria and the ripening conditions [32]. According to Smit *et al*. [16], the flavor components of Gouda cheese are mainly derived from amino acids such as 3-methylbutanal, 3- methylbutanol, methanethiol, dimethylsulphide, 2-methylporpanol and dimethyltrisulphide. Additionally, there are other compounds, such as diacetyl, ketones, aldehydes, and fatty acids. As a result, the tested miniature Gouda cheeses have malty, cabbage, alcoholic, fruity, sweet, buttery, and sulphurous flavors. Methylbutanone and 2- methyl-1-propanol are typically derived from the branched-chain amino acids leucine and valine, respectively [33]. These flavor compounds demonstrated that the miniature cheeses produced by LDTM6802 and LDTM6803 have a characteristic flavor profile, and that starter bacteria play an important role in the characteristic flavor compounds and flavor intensity of cheese.

In this study, starter strains originated from Gangwon Province were assessed for their flavor-forming ability and validated in a miniature Gouda-type cheese model. Generally, Gouda-type cheese is ripened for a length of time ranging from more than two months to several years [32]. Long-term cheese ripening in the laboratory has limits; thus, it is also necessary to shorten the ripening period. Starter bacteria affect flavor formation at least 2 to 3 weeks after cheese production [24]. According to Ur-Rehman *et al*. [24], miniature cheeses require about 28 days for ripening. Long-term experiments with large-scale Gouda-type cheese production are needed to evaluate the actual flavor formation, and the flavor mechanisms by starter bacteria should be further investigated. The evaluation approach in this study can be used to screen starter bacteria with flavor formation ability and can also be applied to further develop Korean cheese technology and industry.

## Supplemental Materials



Supplementary data for this paper are available on-line only at http://jmb.or.kr.

## Figures and Tables

**Table 1:** The summary of in vitro phenotypic and genotypic characteristics of the most promising cheese starter LAB with flavor-forming ability.

Source	Target bacteria	Coagulant activity		pH		Target gene^a^							GDH activity (U)^#^
		
		24h	48h	6h	48h	*adhE*	*bcaT*	*araT*	*estA*	*adh*	*citP*	*kdcA*	
Chr. Hansen (Control)	*Lactococcus lactis* subsp. *lactis* IL1403	-	-	6.06	5.27	+	+	+	+	+	-	+	0.157±0.026
	*Lactococcus lactis* subsp. *lactis* biovar *diacetylactics* CHN-11-1	++	++	6.15	4.39	+	+	+	+	+	+	+	0.151±0.004
	*Lactococcus* *lactis* subsp. *cremoris* CHN-11-3	+	++	6.10	4.57	-	+	-	-	-	-	-	0.134±0.012
	*Leuconotoc pseudomesenteroides* CHN-11-48	-	-	6.05	5.72	-	-	-	-	-	-	-	0.230±0.010**
Cucumber kimchi	*Lactococcus lactis* subsp*. lactis* LDTM6802	+	++	5.76	4.54	+	+	+	+	+	-	-	0.224±0.028*
Raw milk	*Lactococcus lactis* subsp*. cremoris* LDTM6803	++	++	5.27	4.28	-	+	-	-	-	-	+	0.211±0.020

^a^Target gene: *adhE*, bifunctional aldehyde/alcohol dehydrogenase; *bcaT*, branched-chain aminostransferase; *araT*, aromatic aminotransferase; *estA*, esterase A; *adh*, alcohol dehydrogenase; *citP*, citrate permease; *kdcA*, keto acid decarboxylase.

^#^*p*-value were analyzed by one-way analysis of variance (ANOVA) (^*^p ≤ 0.05; ^**^p ≤ 0.01).

**Table 2:** Different flavor profiling and expected sensory descriptors from 10°C ripened miniature cheese by an electronic nose.

Chemical Names		Formula	Sensory descriptors	Reference

Control groups	Test group			
Acetoin	Acetoin	C_4_H_8_O_2_	Butter, Coffee, Creamy	Arochembase^a^
Butane-2,3-dione	Butane-2,3-dione	C_4_H_6_O_2_	Butter, Caramelized, Creamy, Fruity, Pineapple	Arochembase
Ethanol	Ethanol	C_2_H_6_O	Alcoholic, Ethanol, Pungent, Sweet	Arochembase
Formic acid	Formic acid	CH_2_O_2_	Savory	FEMA^b^
1,3-Dimethylcyclohexane	1,3-Dimethylcyclohexane	C_8_H_16_	Perfumery	Arochembase
3-Buten-2-one	3-Buten-2-one	C_4_H_6_O	Sweet	Arochembase
	Enflurane	C_3_H_2_CIF_5_O	Etheral, Pungent	Arochembase
	Isopropyl isothiocyanate	C_4_H_7_NS	Savory	FEMA
	Methylbutanone	C_5_H_10_O	Camphor	Arochembase
	Methyl isothiocyanate	C_2_H_3_NS	Nauseating, Toxic smell	Duque *et al*. 2001
	1-Propanol, 2-methyl-	C_4_H_10_O	Alcoholic, Bitter, Chemical, Glue, Leek, Licorice, Solvent, Winey	Arochembase
	2,4-Dimethylfuran	C_6_H_8_O	Onion, Galic, Leek	Villière *et al*. 2015

^a^Database provided by electronic nose manufacturer (Alpha M.O.S, France) which is linked with NIST database (http://webbook.nist.gov/chemistry).

^b^FEMA: Flavor & Extract Manufacturers Association.
