# Supplementary material for: Investigation of Flavor-Forming Starter Lactococcus lactis subsp. lactis LDTM6802 and Lactococcus lactis subsp. cremoris LDTM6803 in Miniature Gouda-Type Cheeses
Source: J Microbiol Biotechnol. 2020 Jun 5;30(9):1404–11. doi: 10.4014/jmb.2004.04004 (PMC9728400; doi:10.4014/jmb.2004.04004)
Supplement: Supplementary file 1 [file JMB-30-9-1404-supple.pdf]

https://doi.org/10.4014/jmb.2004.04004

Supplement 1. Primers used in this study.

Group	Target gene	Primer	Sequences (5'-3')	TM (℃)	GC (%)	Amplicon (bp)	Reference
1	*adhE*	AAdh_F	CAAGTCGATACTATTGTC	48.0	38	2,158	In this study
		AAdh_R	TTTATGAGCAAGTGAGTG	49.9	38		
	bcaT	BcAT_F	TACCTTTTCGTTATATCGC	49.8	36	884	In this study
		BcAT_R	TCCTACTTCTGTTTCTGAA	49.9	36		
	araT	ArAT_F	GTCATTATACTGGAATGG	47.7	38	750	In this study
		ArAT_R	CAAGGTCTGACATCTTTT	49.0	38		
	estA	Est_F	GGGATGAATCGAAAAGTT	48.8	38	617	In this study
		Est_R	CCTAATTTTTTCAACTCGG	48.0	36		
2	*adh*	Adh_F	TGTTGAAAAGGAACTTCG	48.7	38	880	In this study
		Adh_R	AATTGGTTTTACCTTACCC	48.3	36		
	citP	P4(S)	GGAGTTGGTGCTGGTATTGTG	60.2	52	616	Klijn et al. 1995
		P5(A)	CCAACCCTGCTGTAATAGCAG	59.7	52		
3	*kdcA*	3fw	GTATACAGTAGGACATTACC	51.1	40	1,630	Smit et al. 2005
		1633Rev	GCTCAGCAAATAATTTACCC	51.7	40		

*TM: Melting temperature, GC: guanine-cytosine content

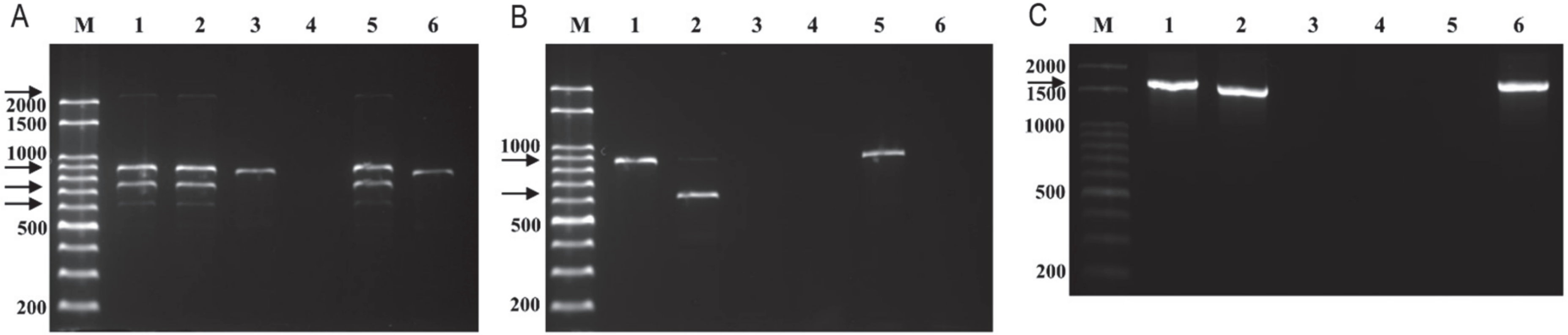

Supplement 2. Agarose gel electrophoretic analysis of multiplex PCR with the group1 (adhE, bcaT, araT, estA) (A), group2 (adh, citP) (B), and group3 (kdcA) (C). M: 100bp ladder, 1: IL1403, 2: CHN-11-1, 3: CHN-11-3, 4: CHN-11-48, 5: LDTM6802, 6: LDTM6803.
